# The treatment methods for post‐stroke visual impairment: A systematic review

**DOI:** 10.1002/brb3.682

**Published:** 2017-04-06

**Authors:** Kerry Louise Hanna, Lauren Rachel Hepworth, Fiona J. Rowe

**Affiliations:** ^1^ Department of Health Services ResearchUniversity of LiverpoolLiverpoolUK

**Keywords:** intervention, management, review, stroke, treatment, visual impairment

## Abstract

**Aim:**

To provide a systematic overview of interventions for stroke related visual impairments.

**Method:**

A systematic review of the literature was conducted including randomized controlled trials, controlled trials, cohort studies, observational studies, systematic reviews, and retrospective medical note reviews. All languages were included and translation obtained. This review covers adult participants (aged 18 years or over) diagnosed with a visual impairment as a direct cause of a stroke. Studies which included mixed populations were included if over 50% of the participants had a diagnosis of stroke and were discussed separately. We searched scholarly online resources and hand searched articles and registers of published, unpublished, and ongoing trials. Search terms included a variety of MESH terms and alternatives in relation to stroke and visual conditions. Article selection was performed by two authors independently. Data were extracted by one author and verified by a second. The quality of the evidence and risk of bias was assessed using appropriate tools dependant on the type of article.

**Results:**

Forty‐nine articles (4142 subjects) were included in the review, including an overview of four Cochrane systematic reviews. Interventions appraised included those for visual field loss, ocular motility deficits, reduced central vision, and visual perceptual deficits.

**Conclusion:**

Further high quality randomized controlled trials are required to determine the effectiveness of interventions for treating post‐stroke visual impairments. For interventions which are used in practice but do not yet have an evidence base in the literature, it is imperative that these treatments be addressed and evaluated in future studies.

## 1 Introduction

Visual impairments following stroke may include abnormalities of central and/or peripheral vision, eye movements and a variety of visual perception problems such as inattention and agnosia. The visual problems (types of visual impairment) can be complex including ocular as well as cortical damage (Jones & Shinton, 2006; Rowe et al., 2009a). Visual impairments can have wide reaching implications on daily living, independence, and quality of life. Links with depression have also been documented in the literature (Granger, Cotter, Hamilton, & Fiedler, 1993; Nelles et al., 2001; Ramrattan et al., 2001; Tsai et al., 2003; West et al., 2002). The estimation of the overall prevalence of visual impairment is approximately 60% at the acute stage following stroke (Ali et al., 2013; Barrett et al., 2007; Clisby, 1995; Freeman & Rudge, 1987; Isaeff, Wallar, & Duncan, 1974; Rowe et al., 2009b; Rowe et al., 2013). A review of the individual prevalence figures and the recovery rates for each of the possible post‐stroke visual impairments has been reported elsewhere in the literature (Hepworth et al., 2016).

In order to treat and manage visual impairments caused by stroke it is important to establish the range and effectiveness of the available treatment options. The aim of this literature review is to provide a comprehensive synthesis of the evidence relating to treatment of visual problems after stroke.

## 2 Methods

We planned an integrative review, aiming to bring together all evidence relating to intervention of stroke‐related visual problems. A detailed protocol was developed prior to the review. This review was carried out as part of a larger synthesis of evidence relating to visual problems after stroke.

### 2.1 Inclusion criteria for considering studies for this review

#### 2.1.1 Types of studies

The following types of studies were included: systematic reviews, randomized controlled trials, controlled trials, cohort studies, observational studies, and retrospective medical note reviews. Case reports were excluded due to the high risk of bias associated with these types of reports. All languages were included and translation obtained.

#### 2.1.2 Types of participants

We included studies of adult participants (aged 18 years or over) diagnosed with a visual impairment as a direct cause of a stroke. Studies which included mixed populations were included if over 50% of the participants had a diagnosis of stroke and data were available for this subgroup. Studies were also included if the participant group comprised of health care professionals who worked with and treated visual impairment problems associated with stroke.

### 2.2 Search methods for identification of studies

We used systematic search strategies to search key electronic databases and contacted known experts in the field.

We searched the Cochrane Stroke Group Trials Register, the Cochrane Eyes and Vision Group Trials Register, and the following electronic bibliographic databases:

1. The Cochrane Central Register of Controlled Trials (CENTRAL) (*The Cochrane Library* September 2015);
2. MEDLINE (1950 to February 2016);
3. EMBASE (1980 to February 2016);
4. CINAHL (1982 to February 2016);
5. AMED (1985 to February 2016);
6. PsycINFO (1967 to February 2016);
7. Dissertations & Theses (PQDT) database (1861 to February 2016);
8. British Nursing Index (1985 to February 2016);
9. PsycBITE (Psychological Database for Brain Impairment Treatment Efficacy, www.psycbite.com). (July 2004 to February 2016)

In an effort to identify further published, unpublished and ongoing trials, we:

1. Searched the following registers of ongoing trials:
  - ClinicalTrials.gov (http://clinicaltrials.gov/);
  - Current Controlled Trials (www.controlledtrials. com);
  - Trials Central (www.trialscentral.org);
  - Health Service Research Projects in Progress (wwwcf.nlm.nih.gov/hsr_project/home_ proj.cfm);
  - National Eye Institute Clinical Studies Database (http://clinicalstudies.info.nih.gov/cgi /protinstitute.cgi?NEI.0.html)
2. Hand‐searched the *British and Irish Orthoptic Journal*,* Australian Orthoptic Journal*, and proceedings of the European Strabismological Association (ESA), International Strabismological Association (ISA), International Orthoptic Association (IOA) (http://pcwww.liv.ac.uk/~rowef/index_files/Page646.htm) and proceedings of Association for Research in Vision and Ophthalmology (www.arvo.org);
3. Performed citation tracking using Web of Science Cited Reference Search for all included studies;
4. Searched the reference lists of included trials and review articles about vision after acquired brain injury;
5. Contacted experts in the field (including authors of included trials, and excluded studies identified as possible preliminary or pilot work).

Search terms included a variety of MESH terms and alternatives in relation to stroke and visual conditions (Table 1).

**Table 1:** Search terms

Cerebrovascular disorders/ Brain ischemia/ Intracranial Arterial Disease Intracranial Arteriovenous Malformations/ Intracranial Embolism and Thrombosis/Stroke/	Eye Movements/ Eye/ Eye Disease/ Visually Impaired Persons/ Vision Disorders/ Blindness/ Diplopia/ Vision, Binocular/ Vision, Monocular/ Visual Acuity/ Visual Fields/ Vision, Low/ Ocular Motility Disorders/ Blindness, Cortical/ Hemianopsia/ Abducens Nerve Diseases/ Abducens Nerve/ Oculomotor Nerve/ Trochlear Nerve/ Visual Perception/ Nystagmus strabismus smooth pursuits saccades depth perception stereopsis gaze disorder internuclear opthalmoplegia Parinaud's syndrome Weber's syndrome skew deviation conjugate deviation oscillopsia visual tracking agnosia hallucinations
OR	OR
	AND

### 2.3 Selection of studies

The titles and abstracts identified in the primary review were independently screened by two authors (FR, LH) using the inclusion criteria discussed previously.

Where it was not possible to establish if a study met these criteria from the title or abstract, the full paper was obtained. A secondary review of the full papers was then undertaken independently by two authors (FR, LH) to determine which studies should be included. In the case of disagreement for inclusion of studies, an option was available to obtain a third author opinion (KH). In practice, this was not required as no disagreements occurred for inclusion of papers.

### 2.4 Data extraction

A pre‐designed data extraction form was designed. Data was extracted and documented by one author (LH) and verified by another (FR).

### 2.5 Quality assessment

Two reviewers (KH and LH) independently reviewed the quality of the studies included in this review using the following four checklists. For the evaluation of the quality of evidence in randomized control and control trials, an adapted version of the CONSORT (Consolidated Standards of Reporting Trials) statement was used. The CONSORT statement covers 25 items within the following domains; title/abstract, introduction, methods, results, discussion, and other information (Moher et al., 2010). An adapted version of the STROBE (Strengthening the Reporting of Observational Studies in Epidemiology) statement was used to assess the quality of cross‐sectional, cohort, and control studies. The STROBE statement covers 22 items from introduction, methods, results, and discussion (Elm et al., 2007). An adapted version of the PRISMA (Preferred Reporting for Systematic reviews and Meta‐Analyses) statement was used to assess quality of evidence in review articles, including the three Cochrane review papers used. This covers 27 items within title, abstract, introduction, methods, results, discussion, and funding (Moher, Liberati, Tetzlaff, & Altman, 2009). Finally, an adapted version of the GRACE (Good Research for Comparative Effectiveness) statement was used for observational studies with comparative effectiveness. This statement covers 11 items within the domains of data and methods. There is no formal scoring system used in this checklist, but it is suggested that if a paper addresses the majority of the checklist items, then it is deemed reliable (Dreyer, Velentgas, Westrich, & Dubois, 2014).

The adapted version of the STROBE statement used in this review included 18 items. Only the information pertinent to quality appraisal of the studies was included. The items excluded were not considered relevant information i.e. the title/abstract, background, setting, and funding. The adapted version of the CONSORT statement included 31 items of relevance.

All domains covered in these checklists are important factors to consider when evaluating the quality of evidence and risk of bias in the reported articles. These domains were graded ‘high risk’, ‘low risk’, or ‘unclear risk’. If it was clear the domain was performed then this would be described as “reported” and would be recorded as having a low risk of bias. If the domain was not included this would be described as “not reported” and deemed a high risk of bias. Insufficient evidence would be labeled as an “unclear” risk.

## 3 Results

**Figure 1:**
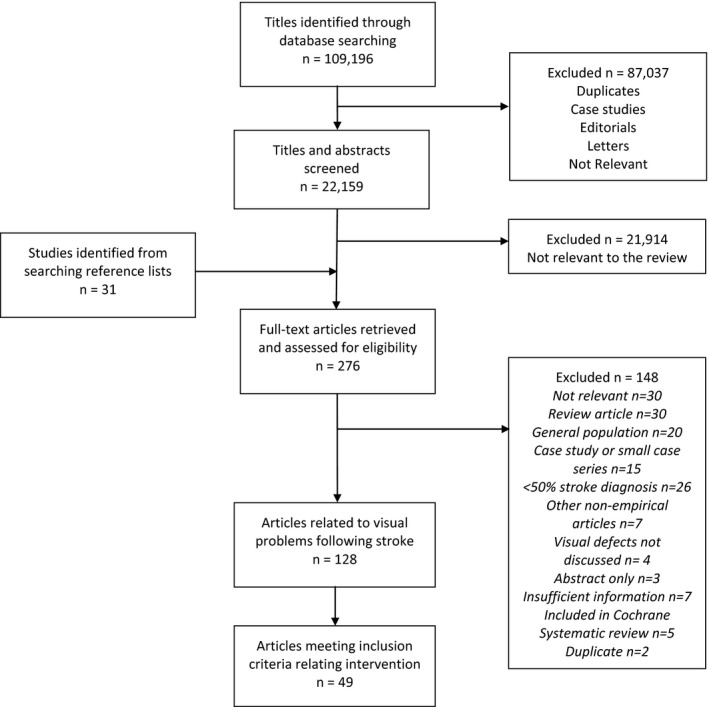
Flowchart of pathway to inclusion of articles

Figure 1 illustrates the results of the search. Forty‐nine articles (3,613 participants and 529 health care professionals) were included. This number includes four Cochrane reviews relating to interventions available for visual problems following stroke. In view of the high standard and rigorous methods of Cochrane reviews, the findings of these four papers are summarized as an overview, followed by a review of trials and studies not included in the Cochrane reviews. Tables 2, 3, 4, 5, 6 display key characteristics of the included studies. The 49 included studies consisted of four Cochrane systematic reviews, seven randomized trials, one randomized crossover trial, two non‐randomized controlled trials, 27 prospective observational studies, three retrospective analysis, four prospective surveys/ questionnaires and one prospective observational study with a questionnaire. One study only used a control group for the pre‐treatment data and so was treated as a prospective observational study and not a controlled trial (Woodhead et al., 2013).

**Table 2:** Results for treatment of visual field defects

Study	Study design	Aim/ objective	Sample size (*n*)	Population	Intervention	Time/ duration of intervention
Aimola et al. (2014)	RCT Parallel design	Evaluate the efficacy and feasibility of an unsupervised reading and exploration computer training	52 Intervention: 28 Control: 24	Mixed Ischemic stroke *n *=* *39, hemorrhage *n *=* *6, TBI *n *=* *6, tumor *n *=* *1 At least 3 months post stroke	Compensatory: Computer based reading and visual exploration training versus sham exploration task	Experimental group = 14 blocks of training per day. Control group = 10 blocks per day. One hour sessions for up to 10 weeks
Bainbridge and Reding (1994) Article taken from cochrane review Pollock et al. (2012a)	RCT	To assess the effect of full field prisms for hemi‐field visual impairments	18	Stroke	Substitutive: 15 ^Δ^ prism versus hemifield prisms	Prism wear while awake for 4 weeks
Bergsma et al. (2012)	Cohort study	Determine whether peripheral training also causes improvement in color and shape perception and reading speed	12	Chronic stroke (6–102 months post stroke)	Restitutive: VRT	40× 1 hr sessions of training, For 10 weeks.
Bowers et al. (2014)	Double masked, multi‐center, randomized crossover trial	Evaluate efficacy of real relative to sham peripheral prism glasses	61	Stroke At least 3 months post stroke	Subsitutive: 57^∆^ prism placed above and below the visual axis versus sham (5^∆^). Horizontal versus oblique positioning	Each set of prisms were worn for 4 weeks. Measured at 6 months
Carter et al. (1983) Article taken from cochrane review Pollock et al. (2012a)	RCT	To test the effect of cognitive skill remediation training versus control/ standard care	33	Stroke With or without visual field defect or neglect	Compensatory: Cognitive skill remediation training	30–40 min 3× weekly for 3–4 weeks
Freeman and Rudge (1987)	Prospective observational study	Identify the Orthoptists’ role in stroke management	76	Stroke	Advice (for field defect and inattention, *n *= 4) occlusion (*n *= 10), prisms (*n *= 7), registered blind (*n *= 2), observation (*n *= 20), glasses (*n *= 5)	Within 1 week post stroke. Follow‐up ranged from 1 week to 4 years
Gall and Sabel (2012)	Prospective non‐controlled trial	Examine whether increased visual functioning after VRT coincides with improved reading abilities	11	Mixed Infarct *n *=* *7, hemorrhage *n *=* *1, AVM *n *=* *1, subarachnoid hemorrhage *n *=* *1, encephalitis *n *=* *1	Restitutive: VRT	30 min 2× daily, 6 days a week, for 6 months
Giorgi et al. (2009)	Cohort study	Evaluate Peli prisms as a low vision optical device for hemianopia in an extended wearing trial	23	Mixed Stroke *n *=* *16, surgery *n *=* *4, TBI *n *=* *2, congenital *n *=* *1	Subsitutive: 40^∆^ prism placed above and below the visual axis	Peli prisms worn for 6 weeks, 3 months and long‐term. “Long‐term” follow‐up not specified
Hayes et al. (2012)	Interventional case series	Evaluate functional changes following the NVT program for homonymous hemianopia after stroke	13	Stroke Within 2 weeks – 6 months post stroke	Compensatory: NVT	One hour per session, 3× per week for 7 weeks
Jacquin‐Courtois et al. (2013)	Prospective observational study	Test the effect of a compensatory eye movement training	7	Mixed Stroke *n *=* *5 Tumor *n *=* *2 Chronic field loss, approx. 2.9 years post stroke	Compensatory: Visual search	1× 30 min session
Jobke et al. (2009) Article taken from cochrane review Pollock et al. (2012a)	Randomized, double blinded, crossover study	To compare extrastriate versus conventional VRT in patients with visual field loss	21	Mixed Stroke/ ischemia *n *=* *10, cranio‐cerebral injury *n *=* *3, brain surgery *n *=* *3, tumor *n *=* *1, meningitis *n *=* *1	Restitutive: Extrastriate VRT versus Conventional VRT	Extrastriate 30 min daily for 90 days. Then crossover of conventional VRT for 90 days
Kasten et al. (1998) Article taken from cochrane review Pollock et al. (2012a)	RCT, double blinded	To assess the effect of computer based training to treat partial blindness	19	Mixed Stroke *n *=* *10, trauma *n *=* *4, other *n *=* *5	Restitutive: VRT	1 hr per day, 6 days per week for 6 months (total = 150 hr)
Kasten et al. (2007) Article taken from cochrane review Pollock et al. (2012a)	RCT	To test the hypothesis that VRT does not benefit from co‐stimulation	23	Mixed stroke, ischemia, cerebral hemorrhage, vascular disease (*n *=* *14 combined), trauma (*n *=* *8), inflammation (*n *=* *1)	Resititutive: Parallel co‐stimulation, moving co‐stimulation or single stimulus	All groups had 30 min 2× daily for 3 months
Lane et al. (2010)	Non‐randomized controlled trial	Explore the efficacy of a visual exploration training	42	Mixed Ischemic *n *=* *28, hemorrhage *n *=* *10, TBI *n *=* *4	Compensatory: Visual exploration training Visual attention training	Exploration training = 40 min sessions, over 2–9 weeks. Attention raining = 30 min sessions, over 2–7 weeks.
Mannan et al. (2010)	Prospective observational study	Characterize changes in eye movements resulting from training	29	Mixed Infarct *n *=* *22, hemorrhage *n *=* *6, surgery *n *=* *1, tumor *n *=* *2 At least 3 months post stroke	Compensatory: Visual search training	20× 40 min sessions for 1 month
Marshall et al. (2010)	Longitudinal cohort	Determine whether visual field expansion occurs with VRT	7	Stroke	Restitutive: VRT using microperimetry	20–30 min 2× daily, 6 days a week, for 3 months
Mazer et al. (2003)	RCT	To compare driving performance after useful field of view retraining (UFOV) compared to traditional visuoperceptual retraining	84	Stroke	Compensatory UFOV versus commercially available computer based visuoperceptual retraining (control)	Both received 20 sessions (each session 30–60 min long) at a rate of 2–4 sessions per week
Mueller et al. (2007)	Prospective observational study	Evaluate the outcome of VRT in a larger sample	302	Mixed Stroke *n *=* *214, trauma *n *=* *43, tumor *n *=* *34, AION *n *=* *5	Restitutive: VRT	1 hr of training, 6 days a week, for 6 months
Nelles et al. (2001)	Prospective observational study	Investigate whether training eye movements would induce change in the neural activity of cortical visual areas	21 Controls: 23 health subjects	Stroke Infarct *n *=* *16 Hemorrhage *n *=* *5	Compensatory: Eyes fixating versus exploratory eye movements	30 min per session, 2× daily, for 4 weeks
Nelles et al. (2010)	Prospective observational study	Can the internet be used as a resource so that suitable patients can build‐up practice to improve	8	Ischemic stroke	Compensatory: Eye movement training	30 min session 1× daily for 4 weeks
Ong et al. (2012)	Longitudinal cohort study	To see if Eye‐search web based hemifield search training improves patients search time and “real world” outcomes	33	Stroke participants with right homonymous hemianopia Infarct *n *=* *14, hemorrhage *n *=* *3, AVM *n *=* *1, unknown *n *=* *15	Compensatory: OKN therapy ‐ “Read right”	20 min of therapy per day (suggested). Patients prompted to test reading speed after 5 hr of therapy accrued.
Ong et al. (2015)	Prospective observational study	Evaluate efficiency of eye movements following visual search training	78	Hemianopic patients with no neglect 77% = stroke patients (8% = tumor, 3% TBI, 13% = other)	Compensatory: Eye‐search scanning exercises online	11 days of therapy (length of each session not specified)
Pambakian et al. (2004)	Prospective observational study	Examine whether directing attention to ARV using a visuospatial cue also increases long‐term neural plasticity	31 (29 completed training)	Mixed Infarct *n *=* *22, hemorrhage *n *=* *6, surgery *n *=* *1, tumor *n *=* *2 At least 3 months post stroke.	Compensatory: Visual search training	20× 40 min sessions, in 1 month
Plow et al. (2010) Article taken from cochrane review Pollock et al. (2012a)	RCT	To test the effect of transcranial direct current stimulation to enhance VRT	8	Stroke	Restitutive: VRT with active tDCS versus VRT with sham tDCS	VRT = 30 min 2× daily for 3 months Active tDCS = 2 mA/ min along with VRT sham tDCS = 30 seconds ramped down to 0 then turned off, along with VRT
Plow et al. (2012)	Pilot, double blinded RCT	Investigate whether training eye movements would induce change in the neural activity of cortical visual areas	12 (8 included in final analysis)	Mixed Stroke *n *=* *10, surgical trauma *n *=* *2 At least 3 months post stroke	Restitutive: VRT compared with active tDCS (control group received sham tDCS)	30 min of training, 3× a week, for 3 months.
Poggel et al. (2004) Article taken from cochrane review Pollock et al. (2012a)	RCT	To assess whether or not attentional cueing improves VRT	20	Mixed post‐genicular lesions	Restitutive: VRT with attentional cueing versus VRT with no attentional cueing	30–35 min 2× daily, for 56 sessions lasting approx. 1 month
Poggel et al. (2007)	Retrospective analysis of a prospective clinical trial. Retrospective analysis of questionnaire	Assess the possible efficacy of tDCS combined with VRT	Trial = 19 questionnaire = 121	Mixed Infarct *n *=* *15, vascular *n *=* *3, TBI *n *=* *1	Restitutive: VRT	30–35 min of training, 2× daily, for 6 months.
Pollock, Hazelton, & Brady (2011a)	Survey	To explore the current assessments, protocols, referrals, and treatments of visual problems after stroke by OTs	55	Occupational therapists	Visual field, eye movement disorders and visual neglect (scanning training, patching/ prisms, ADL training, reading aids/ magnifiers, information, environment modification)	45% of OTs said they would treat within 2 weeks of stroke. 75% said they would treat patients within 6 weeks of stroke. 38% said they would continue treatment up to 3 months
Pollock, Hazleton, & Brady (2011b)	Survey	To explore the current assessments, protocols, referrals, and treatments of visual problems after stroke by Orthoptists	14	Orthoptists	Visual field, eye movement disorders and visual neglect (scanning training, patching/ prisms, ADL training, reading aids/ magnifiers, information, environment modification)	Time of intervention not stated. 86% did not have a protocol/ management plan for visual treatment of stroke patients
Pollock et al. (2012a)	Cochrane systematic review	To determine the effects of interventions for visual field defects after stroke	13 studies *n *=* *344	Mixed Stroke *n *=* *285	Various (studies listed individually)	Resistutive *n *= 5, compensatory *n *= 5, substitutive *n *= 3.
Reinhard et al. (2005)	Prospective observational study	Examine if VRT is able to change absolute homonymous field defects	17	Mixed Ischemia *n *=* *11, trauma/surgery *n *=* *4, hemorrhage *n *=* *2	Restitutive: VRT using scanning laser ophthalmoscope	1 hr of training, 6× per week, for 6 months.
Romano et al. (2008)	Retrospective analysis	Determine the effect of a visual rehabilitation intervention on visual field defects	161	Mixed stroke 84%, TBI 9%, surgery 3%, other/unknown 4%	Restitutive: VRT	30 min of training, 6 days per week, for 26–30 weeks.
Rossi et al. (1990) Article taken from cochrane review Pollock et al. (2012a)	RCT	To see if Fresnel prisms improve visual perception	30	Stroke	Substitutive: 15 dioptre hemi‐circular Fresnel prisms applied to glasses along with standard rehabilitation	Worn all day for 4 weeks
Roth et al. (2009) Article taken from cochrane review Pollock et al. (2012a)	RCT	Comparing explorative saccade and flicker training	30	Mixed stroke/ hemorrhage *n *=* *26, other *n *=* *4	Compensatory: exploratory eye scanning training Restitutive: flicker‐stimulation training	Both = 30 min 2× daily, 5 days a week for 6 weeks
Rowe et al. (2009a)	Prospective multicenter cohort trial	To profile the site of stroke, type and extent of field loss, treatment and outcome	915 *n *=* *479 with field loss *n *=* *151 with field loss as only complaint	Stroke	Compensatory: typoscope, orthoptic exercises, advice (awareness of visual field loss, reading strategies, scanning eye and head movements, use of lighting, compensatory head posture, and registration for visual impairment) Substitutive: Peli prisms, diplopia prisms, occlusion, low vision aids	Follow‐up between 2 weeks and 3 months Duration of individual treatments not specified
Sabel et al. (2004)	Prospective observational study	Evaluate the efficacy of VRT using different perimetry methods	16	Mixed Ischemia *n *= 11 Surgery *n *= 3 Hemorrhage *n *= 2 At least 15 months post stroke	Restitutive: VRT measured with different methods of perimetry: Tubinger, automated and scanner laser ophthalmoscope	Between 30 – 60 min per session, and performed between daily – 6 weeks
Sabel et al. (2013)	Prospective observational study	Investigate the role of residual vision in recovery	23	Stroke ‐ at least 1 month post stroke	Restitutive: VRT	6 months of training (length and duration of training sessions not explained)
Schmielau and Wong (2007)	Cohort study	To evaluate whether restoration of VF in patients with homonymous hemianopia is possible using the LRP	20	Mixed Infarction *n *=* *11, hemorrhage *n *=* *7, trauma *n *=* *2	Restitutive: VRT using the Lubeck reaction perimeter	45 min of training, 2× a week. Average length of training = 8.2 months (range = 2–27 months)
Spitzyna et al. (2007) Article taken from cochrane review Pollock et al. (2012a)	RCT	To see if optokinetic therapy improves test reading for hemianopic dyslexia	22	Mixed	Compensatory: optokinetic nystagmus inducing reading therapy	4 weeks of training (minimum of 400 min of rehabilitation) 20× 20 min sessions
Szlyk et al. (2005) Article taken from cochrane review Pollock et al. (2012a)	Randomized crossover design	To assess the use of prisms for navigation and driving for patients with hemanopia	10	Mixed population injury involving occipital lobe only	Sustitutive: Gottlieb visual field awareness system 18.5 dioptre lens versus 20 ^Δ^ Fresnel prisms	VFAS = training of 4× 2–3 hr indoor sessions with LVA specialist and 8× 2 hr outdoor sessions behind the wheel Prisms were worn for 3 months
Weinberg et al. (1977) Article taken from cochrane review Pollock et al. (2012a)	RCT	To test the effect of visual scanning training on reading related tasks	57	Stroke	Compensatory: visual scanning training	1 hr a day for 4 weeks (20 hr of training)
Weinberg et al. (1979) Article taken from cochrane review Pollock et al. (2012a)	RCT	To test the effect of visual scanning training on reading related tasks	53	Stroke	Compensatory: visual scanning training	1 hr a day for 4 weeks (20 hr of training)
Zihl and von Cramon (1979)	Prospective observational study	Present evidence that diminished visual function can be improved by systematic stimulation of impaired areas of the visual field.	12	Mixed Infarct *n *=* *6, hemorrhage *n *=* *2, tumor *n *=* *3, hypoxia *n *=* *1	Restitutive: VRT	1 hr of training per day. Total length of treatment not specified
Zihl and von Cramon (1982)	Prospective observational study	To test the hypothesize that recovery takes place at the level of the striate cortex	30	Mixed Vascular *n *=* *24, surgery *n *=* *6	Comparing restitutive VRT and compensatory eye movement training: Light detection versus Saccadic localization	Treatment started between 1–6 months of onset of field defect. Total length of treatment not specified.
Zihl and von Cramon (1985)	Retrospective case series (from a larger study)	To assess the recovery of visual field loss with VRT versus compensatory eye movement training	55 post hoc sample from *n *=* *125	Mixed 80% Infarct 20% TBI At least 4 weeks post stroke	Compensatory: Exploratory visual search	Training performed between daily‐ 3× weekly. Total length of treatment not specified. Followed up for at least 4 months post treatment
Zihl (1995)	Retrospective analysis	Investigate eye movement patterns in patients with hemianopic dyslexia	*n *=* *50 before treatment assessment *n *=* *20 after treatment assessment	Stroke 3–12 weeks post stroke	Compensatory: Optokinetic therapy	Not specified

Articles taken from Cochrane reviews are included in this table for information only and are not included in the overall review.

**Table 3:** Results for treatment of visual neglect/ inattention

Study	Study design	Aim/ objective	Number of participants	Type of population	Intervention	Time/ duration of intervention
Beis et al. (1999)	RCT	Compare control with occlusion	*n *= 22	Right sided vascular lesion. 42–56 days post stroke.	Half eye patches versus full eye patches	Glasses with occlusion were worn 12 hr a day for 3 months
Bowen et al. (2013)	Cochrane systematic review	Assess whether cognitive rehabilitation improved neglect	23 studies *n *= 628	Stroke	Top‐down approaches Bottom‐up approaches Mixed approaches	Various dependant on intervention type (4 days–2 months)
Cherney et al. (2002) Article taken from cochrane review Bowen et al. (2013)	RCT	A comparison of two approaches to treat unilateral neglect (top down approach)	*n *= 4	Stroke Right hemisphere	Visual scanning, practising letter and word cancellation tasks versus repetitive practise of functional task/ oral reading	Both groups = 20 sessions Frequency of sessions unknown
Cottam (1987) Article taken from cochrane review Bowen et al. (2013)	RCT	Assessing visual scanning training for left hemispatial neglect (top down approach)	*n *= 12	Stroke	Visual scanning in three separate phases: Scanning a light board when stationary, while self‐propelling, and naming objects present on both sides	Each phase = 5× 5 hr sessions (5 days)
Datié et al. (2006)	Prospective observational study	Investigate the use of prisms for neglect	*n *= 20 patients *n *= 15 healthy volunteers	Unilateral vascular lesion with left sided neglect	Prisms	15 min of prism adaptation
Edmans et al. (2000) Article taken from cochrane review Bowen et al. (2013)	RCT	To compare the effectiveness of the transfer of training and functional approaches in improving perceptual and functional abilities after stroke (top down approach)	*n *= 42	Stroke	Cueing and feedback teach compensation versus functional approaches	Both groups = 2.5 hr of training per week for 6 weeks
Fanthome et al. (1995)	RCT	The treatment of neglect using feedback eye movements (top down approach)	*n *= 18	Stroke Right hemispheric	Specially adapted glasses with auditory signal versus no treatment	2 hr 40 min per week for 4 weeks
Ferreira et al. (2011) Article taken from cochrane review Bowen et al. (2013)	RCT	To compare mental practice versus visual scanning to treat neglect (top down approaches)	*n *= 10	Stroke Right hemispheric	Visual scanning versus mental practice	10× 1 hr sessions over 5 weeks
Fong et al. (2007) Article taken from cochrane review Bowen et al. (2013)	RCT	To assess the effect of trunk rotation with and without hemifield eye patching to treat neglect (bottom up approach)	*n *= 60	Stroke	Voluntary trunk rotation versus Trunk rotation with hemi field eye patching versus conventional OT (control)	Trunk rotation = 1 hr per day (15 min ADLs and 45 min trunk rotation) for 5 day per week for 30 days (30 hr)
Freeman and Rudge (1987)	Prospective observational study	Identify the orthoptic problems associated with stroke	*n *= 76	Stroke	Advice (for field defect and inattention, *n *= 4) occlusion (*n *= 10), prisms (*n *= 7), registered blind (*n *= 2), observation (*n *= 20), glasses (*n *= 5)	Within 1 week post stroke. Follow‐up ranged from 1 week to 4 years
Kalra et al. (1997) Article taken from cochrane review Bowen et al. (2013)	RCT	To evaluate the effectiveness of spatial cueing during motor activity on functional outcome and resource use in neglect patients (bottom up approach)	*n *= 50	Stroke	Conventional therapy versus spatial‐motor cueing	47.7 hr of conventional therapy over 64 days versus 27.8 hr of therapy with spatial‐motor cueing over 36 days
Kerkhoff et al. (2012) Article taken from cochrane review Bowen et al. (2013)	RCT	To compare the effect of OKS (bottom up) and visual scanning training (top down) in the treatment of neglect	*n *= 6	Stroke	Optokinetic stimulation (OKS) versus Visual scanning training	Both = 20× treatment sessions for 50 min, 5 sessions per week
Kerkhoff et al. (2013)	RCT	Compare the effects of smooth pursuit eye movement therapy on auditory and visual neglect in chronic stroke patients	*n *= 50	Stroke Ischemia *n *= 37 Hemorrhage *n *= 8 All had left‐sided visual and auditory neglect. At least 1 month post stroke	Smooth pursuit eye movement training *n *= 24 versus Visual scanning training *n *= 21	5× 50 min sessions, over period of 7–9 days.
Luukkainen‐Markkula et al. (2009) Article taken from cochrane review Bowen et al. (2013)	RCT	Comparing visual scanning training (top down) and arm activation training (bottom up)	*n *= 12	Stroke	Visual scanning training versus left arm activation training	Arm activatio*n *= 20–30 hr of left arm activation Visual scanning = 1 hr 4× weekly (10 hr) with OT training 1 hr 2× daily both groups = 48 hr of treatment in 3 weeks
Machner et al. (2012)	RCT	To establish if hemifield eye patching or OKS is an effective therapy for neglect in acute stroke patients	*n *= 21	Acute right hemispheric stroke patients	Hemifield eye patching and optokinetic stimulation therapy	OKS = 15 min sessions daily for one month. Eye patch to be worn full time.
Menon‐Nair et al. (2007)	Survey	To obtain a response from 61 stroke inpatients	*n *= 663	Occupational Therapists	Perceptual training, scanning training, activation treatment, cognitive therapy, eye patch, constraint‐induced therapy, prisms, trans‐electrical nerve stimulation	Not specified
Mizuno et al. (2011) Article taken from cochrane review Bowen et al. (2013)	RCT, multi center, double blinded	Comparing search training with and without prisms (bottom up approach)	*n *= 38	Stroke	Training = pointing at targets whilst sitting – 30× without prisms, 90× with, then 60× without Prisms shift field 12̊ right	2× daily 20 min sessions, 5 days a week for 2 weeks (20 sessions)
Nys et al. (2008) Article taken from cochrane review Bowen et al. (2013)	RCT, single blinded	To assess the effect of prism adaptation on neglect rehabilitation (bottom up)	*n *= 16	Stroke	Prism adaptation	30 min sessions for 4 days in a row versus placebo
Polanowska et al. (2009) Article taken from cochrane review Bowen et al. (2013)	RCT, double blinded	To assess the effectiveness of left hand stimulation bottom up) combined with scanning training (top down) to treat neglect	*n *= 40	Stroke	Electrical somatosensory stimulation to left hand with conventional visual scanning training	45 min per sessions for 5 days weekly for 1 month (20 sessions)
Pollock, Hazelton, & Brady, (2011a)	Survey	To explore the surrent assessments, protocols, referrals, and treatments of visual problems after stroke by OTs	*n *= 55	Occupational Therapists	Visual field, eye movement disorders and visual neglect (scanning training, patching/ prisms, ADL training, reading aids/ magnifiers, information, environment modification)	45% of OTs said they would treat within 2 weeks of stroke. 75% said they would treat patients within 6 weeks of stroke. 38% said they would continue treatment up to 3 months
Pollock, Hazleton, & Brady (2011b)	Survey	To explore the current assessments, protocols, referrals, and treatments of visual problems after stroke by Orthoptists	*n *= 14	Orthoptists	Visual field, eye movement disorders, and visual neglect (scanning training, patching/ prisms, ADL training, reading aids/ magnifiers, information, environment modification)	Time of intervention not stated. 86% did not have a protocol/ management plan for visual treatment of stroke patients
Robertson (1990) Article taken from cochrane review Bowen et al. (2013)	RCT	To assess the effect of microcomputer based rehabilitation on left sided visual neglect (top down)	*n *= 30	Stroke	Computerized scanning and attention training versus Recreational computing	14× 75 sessions, 2× weekly for 7 weeks (15 ½ hr) versus 11.4 hr of recreational computing
Robertson et al. (2002) Article taken from cochrane review Bowen et al. (2013)	RCT	To explore whether or not limb activation rehabilitation reduces left sided motor impairment in neglect patients (bottom up)	*n *= 40	Stroke	Wearing a limb activation device during perceptual training versus Perceptual training with inactive limb device	45 min training per week for 12 weeks
Rossi et al. (1990) Article taken from cochrane review Bowen et al. (2013)	RCT	To assess the use of Fresnel prisms to improve visual perception (bottom up approach)	*n *= 39	Stroke	15 ^Δ^ base out hemi‐field prism versus placebo	Worn for all daytime activities
Rusconi et al. (2002) Article taken from cochrane review Bowen et al. (2013)	RCT	To investigate the effect of cueing on visual scanning therapy to treat neglect (top down)	*n *= 24	Stroke	Visual scanning with and without verbal and visuospatial cueing	5× 1 hr sessions per week for 2 consecutive months (40 sessions)
Schroder et al. (2008) Article taken from cochrane review Bowen et al. (2013)	RCT	A comparison of visual exploration training with and without OKN in the treatment of neglect (combined = bottom up, scanning alone = top down)	*n *= 30	Stroke	Visual exploration versus Visual exploration and OKS	Both = 20× 25–40 min sessions over 4 weeks
Tsang et al. (2009) Article taken from cochrane review Bowen et al. (2013)	RCT	To investigate the efficacy of right half‐field eye patching in treating subacute stroke patients with neglect trial. (bottom up)	*n *= 35	Stroke	Conventional OT training with or without half‐field eye patching (right sided)	5× 60 min OT sessions per week, with or without hemifield eye patching worn for an average 12 hr daily for 4 weeks
Turton et al. (2010) Article taken from cochrane review Bowen et al. (2013)	RCT, single blinded	To assess if prism adaptation therapy helps improve self‐care in stroke patients (bottom up)	*n *= 37	Stroke	Prism adaptation training (10^Δ^) with repeated pointing movements to targets	Training once a day each working day for 2 weeks
Weinberg et al. (1977) Article taken from cochrane review Bowen et al. (2013)	RCT	To test the effect of visual scanning training on reading related tasks (top down)	*n *= 57 (25/ 57 reported on as severe data)	Stroke	Visual scanning training	1 hr a day for 4 weeks (20 hr of training)
Welfringer et al. (2011) Article taken from cochrane review Bowen et al. (2013)	RCT	The use of visuomotor imagery in neglect rehabilitation (top down)	*n *= 30	Stroke	Visuomotor‐imagery therapy	2× 30 min sessions daily for 3 weeks (28–30 sessions overall)
Wiart et al. (1997) Article taken from cochrane review Bowen et al. (2013)	RCT	Trunk rotation and scanning treatment for the rehabilitation of stroke patients with neglect (top down)	*n *= 22	Stroke	Experimental treatment with traditional rehabilitation versus Traditional rehabilitation alone	One hour daily for 20 days

Articles taken from Cochrane reviews are included in this table for information only and are not included in the overall review.

**Table 4:** Results for treatment of ocular motility defects

Study	Study design	Aim/ objective	Number of participants	Type of population	Intervention	Time/ duration of intervention
Choudhuri et al. (2007)	Survey	Determine current management of acquired nystagmus by ophthalmologists and neurologists	*n *= 312 ophthalmologists *n *= 148 neurologists	Ophthalmologists and neurologists	Pharmacological Surgical	Not specified
Freeman and Rudge (1987)	Prospective observational study	Identify the orthoptic problems associated with stroke	*n *= 76 excluded = TIA and other medical conditions	Stroke	Advice (for field defect and inattention, *n *= 4) occlusion (*n *= 10), prisms (*n *= 7), registered blind (*n *= 2), observation (*n *= 20), glasses (*n *= 5)	Within 1 week post stroke. Follow‐up ranged from 1 week to 4 years
Leigh et al. (1991) Article taken from cochrane review Pollock et al. (2011)	Randomized double blinded crossover trial	To compare the effect of trihexyphenidyl 5 mg versus tridihexethyl chloride 25 mg on acquired nystagmus	*n *= 10	Mixed (stroke *n *= 2)	Trihexyphenidyl 5 mg (DrugA) versus tridihexethyl chloride 25 mg (Drug B)	Both drugs = 1 capsule per day. Drug dosage increased by 1 tablet per week until patient is taking 4 tablets per day. 1–2 week washout, then drug crossover
Pollock, Hazelton, & Brady, (2011a)	Survey	To explore the current assessments, protocols, referrals and treatments of visual problems after stroke by OTs	*n *= 55	Occupational Therapists	Visual field, eye movement disorders and visual neglect (scanning training, patching/ prisms, ADL training, reading aids/ magnifiers, information, environment modification)	45% of OTs said they would treat within 2 weeks of stroke. 75% said they would treat patients within 6 weeks of stroke. 38% said they would continue treatment up to 3 months
Pollock, Hazleton, & Brady (2011b)	Survey	To explore the current assessments, protocols, referrals, and treatments of visual problems after stroke by Orthoptists	*n *= 14	Orthoptists	Visual field, eye movement disorders and visual neglect (scanning training, patching/ prisms, ADL training, reading aids/ magnifiers, information, environment modification)	Time of intervention not stated. 86% did not have a protocol/ management plan for visual treatment of stroke patients
Pollock et al. (2011)	Cochrane systematic review	Determine the effects of interventions for eye movement disorders	2 studies *n *= 28	2 studies with mixed population *n *= 28 (stroke *n *= 5)	Pharmacological	Not specified
Rowe et al. (2011a)	Prospective observational cohort	Determine prevalence of ocular motor cranial nerve palsies	*n *= 915 (*n *= 89 with cranial nerve palsy)	Stroke	Occlusion (*n *= 30), prisms (*n *= 30), advice (*n *= 59), compensatory mechanisms	Treatment offered after approx. 22 days (0–2,543 days) Duration of individual treatments not specified Only half followed up for review
Rowe et al. (2013b)	Prospective observational cohort	To evaluate the profile of ocular gaze abnormalities occurring following stroke	*n *= 915 (*n *= 207 with gaze abnormalities)	Stroke	Occlusion (*n *= 40), prisms (*n *= 27), refraction (*n *= 22), orthoptic exercises (*n *= 1), advice (*n *= 69)	37 discharged after initial assessment and treatment. 29 referred onto ophthalmology service. 141 offered review appointments (28 did not attend). Follow‐up lasted 2 weeks – 6 months Duration of individual treatments not specified
Strupp et al. (2003) Article taken from cochrane review Pollock et al. (2011)	Prospective RCT, double blinded, crossover.	Assessing the effect of 3,4 diaminopyridine (DAP) on downbeat nystagmus	*n *= 18	Mixed (stroke *n *= 3)	3,4 diaminopyridine (DAP) and lactose 20 mg versus placebo lactose capsule	1 capsule taken Eye movements measured 30 min after taking capsule. Questionnaire undertaken 30 and 60 min after taking capsule.

Articles taken from Cochrane reviews are included in table for information only and are not included in the overall review.

**Table 5:** Results for treatment of central visual impairment

Study	Study design	Aim/Objective	Number of participants	Type of population	Intervention	Time/ duration of intervention
Beasley and Davies (2013)	Randomized crossover study	Consider the use of spectral filters on visual search in stroke patients	*n *= 17	Stroke	Spectral filters and visual search training	2 weeks using the filters. 2 weeks washout. 2 weeks of using placebo filters
Freeman and Rudge (1987)	Prospective observational study	Identify the orthoptic problems associated with stroke	*n *= 76 excluded = TIA and other medical conditions	Stroke	Registered blind (*n *= 2), observation (*n *= 20), glasses (*n *= 5)	Within 1 week post stroke. Follow‐up ranged from 1 week to 4 years
Lotery et al. (2000)	Prospective Observational	Examine visual status of patients after stroke	*n *= 77	Stroke	Glasses	Within 2 weeks of admission with stroke
Pollock, Hazelton, & Brady, (2011a)	Survey	To explore the current assessments, protocols, referrals, and treatments of visual problems after stroke by OTs	*n *= 55	Occupational Therapists	Visual field, eye movement disorders and visual neglect (scanning training, patching/ prisms, ADL training, reading aids/ magnifiers, information, environment modification)	45% of OTs said they would treat within 2 weeks of stroke. 75% said they would treat patients within 6 weeks of stroke. 38% said they would continue treatment up to 3 months
Pollock et al. (2012b)	Cochrane systematic review	Determine if interventions for age‐related visual problems improve functional ability following stroke	0 studies found	–	–	–
Rowe & VIS (2011)	Prospective multicenter cohort	To identify all patients referred with suspected visual impairment who had reported reading difficulty to establish the prevalence of ocular and non ocular causes	*n *= 915 (*n *= 177 with reading difficulty)	Stroke	Advice, reading strategies, typoscopes, low vision aids, occlusion, prisms, exercises, CVI registration.	Review appointments within 3 months. Duration of individual treatments not specified

**Table 6:** Results for treatment of visual perceptual defects

Study	Study design	Aim/Objective	Number of participants	Type of population	Intervention	Time/ duration of intervention
Rowe et al. (2011b)	Prospective observational cohort	Evaluate prevalence of perceptual deficits post stroke	*n *= 178	Stroke	Advice, compensatory strategies, scanning strategies, general awareness	Average = 22 days post stroke (range = 0–2,543 days) duration of individual treatments not specified
Woodhead et al. (2013)	Prospective observational study – repeated measures	Test the efficacy of audio‐visual reading training	*n *= 9	Mixed Infarct *n *= 7 Hemorrhage *n *= 1 TBI *n *= 1 Patients had pure alexia	Audio‐visual reading training. Cross modal word recognition training	Duration of training not stated, follow‐up at 2 and 4 weeks post training

The included articles reported on interventions for one or a combination of two or more visual impairments. Thirty‐three studies (2,233 participants and 69 health care professionals) reported on interventions for visual field loss (Table 2). Nine reported on interventions for visual inattention/ neglect (227 participants and 732 health care professionals, Table 3). Seven of the studies (1,029 participants and 529 healthcare professionals) reported on intervention for ocular motility or alignment defects (Table 4). Six studies (1,085 participants and 55 healthcare professionals) reported on intervention for reduction of central vision (Table 5) and two (187 participants) reported on interventions for visual perceptual defects (Table 6).

### 3.1 Quality of the evidence

A total of 49 articles were included in this review paper and the quality of evidence was assessed for each (Tables S1–S4). Evidence was deemed to be of good quality if the article reported ≥75% of the items on the relevant assessment checklist. Overall, nine of the reported articles scored 100% in the quality of evidence assessment. Thirty‐four out of the 49 articles included in this review reported between 75 and 99% of the checklist items assessed and were deemed to have good quality. Five reported between 50 and 74% of the items. The remaining one article failed to reach 50%, achieving 26% respectively (Zihl & von Cramon, 1979).

## 4 Interventions

### 4.1 Visual field loss

Visual field loss can affect the peripheral and/ or central field of vision following stroke although, less frequently, the central visual field may present as an isolated defect. Visual field defects can often present with visual perceptual disorders, such as visual inattention and / or agnosia, further complicating the treatment of the visual field loss. One Cochrane review relating to visual field loss following stroke focused on three types of interventions: restitutive, compensatory and substitutive (Pollock et al., 2012a). Functional ability in performing activities of daily living was used as a primary outcome measure. Thirteen trials were identified as meeting the inclusion criteria (Bainbridge & Reding, 1994; Carter, Howard, & O'Neil, 1983; Jobke, Kasten, & Sabel, 2009; Kasten, Bunzenthal, Muller‐Oehring, Mueller, & Sabel, 2007; Kasten, Wurst, Behrens‐Baumen, & Sabel, 1998; Plow et al., 2010; Poggel, Kasten, & Sabel, 2004; Rossi, Kheyfets, & Reding, 1990; Roth et al., 2009; Spitzyna et al., 2007; Szlyk, Seiple, Stelmack, & McMahon, 2005; Weinberg et al., 1977, 1979). Limited meta‐analyses were possible and were only completed for compensatory interventions. A key finding was the limited evidence for all interventions related to visual field loss following stroke. It was not possible to comment on the effectiveness of restitutive or substitutive interventions. Pollock, Hazleton, & Brady (2011b) reported that at least half of Orthoptists in Scotland provided typoscopes, Peli prisms, reading aids and scanning therapy to stroke patients with field loss, with advice on head postures and general information being the most frequently reported strategy. Concurrently, Rowe et al. (2013a) reported that advice and raising awareness of the field loss were the most common forms of treatment (52.7%). Advice included reading strategies, scanning eye and head movements, use of lighting, compensatory head posture, and registration for visual impairment. Further treatments of field loss included typoscopes (43.9%) and Peli prisms (28.6%) (Rowe et al., 2013a).

#### 4.1.1 Compensatory treatment

A variety of different visual scanning and search training methods have been reported in the literature. These include computer and paper‐based search and scanning training programmes and use of word search games. They aim to facilitate the patient in learning to compensate for difficulties by improving the speed and accuracy of eye movements made into the visual field defect side. A number of studies have explored the effect of scanning eye movements into the affected visual field. In a study attempting to regain driving ability in hemianopic stroke survivors (Mazer et al., 2003), there were no significant differences in improved driving performance between those undertaking the useful field of view attention retraining programme (UFOV) and those receiving general computer‐based training. In the Cochrane review on interventions for visual field loss (Pollock et al., 2012a), a recommendation was reached that compensatory interventions were more favorable than a placebo or control at improving specific tasks but not at aiding recovery of the visual field.

Expansion of the field by 1–48 degrees has been reported (Zihl & von Cramon, 1985), however, expansion of the visual field due to natural recovery early after stroke onset cannot be ruled out. Specific improvements, however, relate more to speed and accuracy of eye movements into the affected visual field after training with increased reaction times (Aimola et al., 2014; Jacquin‐Courtois, Bays, Salemme, Leff, & Husain, 2013; Lane, Smith, Ellison, & Schenk, 2010; Ong et al., 2012, 2015; Pambakian, Mannan, Hodgson, & Kennard, 2004) and increased number of saccades into the blind field (Mannan, Pambakian, & Kennard, 2010) with some training available freely e.g. Eye‐search (www.eyesearch.ucl.ac.uk) and Read‐right (www.readright.ucl.ac.uk) (Ong et al., 2015). Subjective improvements in ADL, such as reading speed and accuracy, have also been reported by participants (Aimola et al., 2014; Hayes, Chen, Clarke, & Thompson, 2012; Jacquin‐Courtois et al., 2013; Nelles et al., 2001; Ong et al., 2015).

Nelles et al. (2010) reported that such training was associated with increased activity in the ipsilateral cortex to the insult after training with reports that training is task specific. Eye search training improves eye scanning into the affected side with little objective improvement in reading, whilst reading training improves reading ability with little objective improvement on visual search (Ong et al., 2012; Zihl, 1995). In a recent trial, combined training resulted in an improvement in both eye search and reading (Aimola et al., 2014).

Other compensatory interventions listed in the literature are the use of typoscopes, rulers, and vertical reading. Vertical reading was initially mentioned in the literature as an anecdotal report by a patient describing this as helpful with their hemianopia (Wang, 2003). It has since been stated as a rehabilitation option in review articles but no empirical evidence has been published (Sabel & Trauzettel‐Klosinski, 2005; Schuett, 2009; Trauzettel‐Klosinski, 2010).

An ongoing randomized controlled trial acknowledged in the above Cochrane review is currently comparing compensatory intervention (visual search training), substitutive intervention (Peli prisms) and standard care in the form of verbal and written advice, for the treatment of hemianopia following stroke (Rowe, Barton, et al., 2014). The results are yet to be reported but intend to provide a comparison of the above treatments with regard to effectiveness.

#### 4.1.2 Substitutive treatment

Peli prisms use one or two high strength prisms, placed above and/or below the pupil, with the prism base out on the spectacle lens to the side of visual field loss (Peli, 2000). These prisms create a shift of images on the side of the visual field loss so they move to overlay on the seeing field. This in turn acts as a cue for the patient to look toward the affected side.

In a study of Peli prisms, Giorgi, Woods, and Peli (2009) found that the majority (74%) of participants wearing Peli prisms reported a positive difference over six weeks. Of these, 93% continued to wear the prisms for up to three months and 42% at an unspecified ‘long‐term follow‐up’. However, there were no changes to participant responses in the quality of life questionnaire (NEI VFQ‐25) completed over the initial six week period. In a subsequent trial Bowers, Keeney, and Peli (2014) investigated the efficacy of real Peli prisms (57^∆^) versus sham Peli prisms (5^∆^), and further compared horizontal versus oblique positioning of the prisms. Sixty one percent continued prism wear with an equal number from the oblique and horizontal position groups. A significantly higher proportion wished to continue wearing the real prisms with the most common reason being that prisms helped when walking (92%). However, the analysis of this study demonstrated a possible period effect as the participants were aware they would switch to a second prism. As a result, only 12% reported that they would continue to wear the first prism until they had made a comparison with the second, rather than a comparison against no prisms. Forty‐four percent continued wear after trialling the second prism (Bowers et al., 2014).

#### 4.1.3 Visual restoration treatment

Visual restoration therapy (VRT) involves presenting light stimuli at the border area of visual field loss (Pollock et al., 2012a). One key difference between reported studies is the amount of training prescribed. Some studies (*n *=* *7) prescribed a set amount of training for the whole cohort and others had allowed a range in the amount of training completed by their participants (*n *=* *6). Not one of the studies prescribed exactly the same amount of training, rendering it difficult to make direct comparisons.

Three studies prescribed specific session length and number per week but did not specify the total length of treatment (Schmielau & Wong, 2007; Zihl & von Cramon, 1979, 1982). Across these studies, the mean reported expansion of the visual field border ranged from 1 to 11.3 degrees. Eye movement recordings were not undertaken and thus improvement in the visual field due to eye movements could not be excluded.

The majority of studies (*n *=* *7) prescribed variable session lengths and numbers. The length of session varied from 30 min to 1 hr for around six months of training (Gall & Sabel, 2012; Mueller, Mast, & Sabel, 2007; Poggel et al., 2007; Reinhard et al., 2005; Romano, Schulz, Kenkel, & Todd, 2008; Sabel, Kenkel, & Kasten, 2004; Sabel, Kruse, Wolf, & Guenther, 2013). The shorter sessions were repeated more than once per day, adding up to a possible maximum per day commitment of 70 min. The frequency of training varied between six times per week and daily.

A number of studies reported expansion of the visual field following treatment (Bergsma, Elshout, van der Wildt, & van den Berg, 2012; Mueller et al., 2007; Romano et al., 2008). However, for studies in which fixation was controlled and assessed using the scanning laser ophthalmoscope, little or no change in the visual field area was noted (Marshall, Chmayssani, O'Brien, Handy, & Greenstein, 2010; Reinhard et al., 2005; Sabel et al., 2004). Despite little or no improvement in the visual field area, patients reported an improvement in quality of life and ADL, such as mobility and reading (Bergsma et al., 2012; Gall & Sabel, 2012; Mueller et al., 2007; Plow, Obretenova, Fregni, Pascual‐Leone, & Merabet, 2012; Sabel et al., 2004). Although not statistically significant, reports of visual hallucination or less dense areas of visual field loss were also more likely to show improvement (Poggel et al., 2007; Sabel et al., 2013). The majority of studies recruited patients with chronic homonymous hemianopia (longer than six months post onset). Recruitment within three to six months post‐stroke could not rule out natural recovery (Mueller et al., 2007). Thus, subjective improvements noted by patients are more likely to represent adaptation to the visual field defect.

Although a variety of interventions exist for the treatment of visual field loss, not enough high quality research exists to decipher the true efficiency of a number of these treatment options. The current recommendation would be for compensatory strategies to treat post‐stroke visual field loss. Future, longitudinal studies would need to control for spontaneous recovery of visual field loss when determining the validity of restitutive treatments.

### 4.2 Strabismus and ocular motility

Strabismus pertains to misalignment of the two eyes such that one eye does not point in the same direction as the fellow eye. Ocular motility abnormalities can relate to ocular cranial nerve palsies, gaze palsies, nystagmus, and vergence disorders. There are several extensively used interventions for the treatment of various ocular motility problems in mixed etiology populations such as prisms and occlusion/patching. Many interventions have been tested on non‐stroke populations, as the ocular motility defects that arise as a result of stroke can also be caused by other neurological conditions.

#### 4.2.1 Pharmacology treatment

A Cochrane review relating to eye movement defects following stroke focused solely on pharmacologic interventions for nystagmus, as no trials relating to restitutive, compensatory or substitutive treatments were found specifically for stroke populations with other ocular motility disorders (Pollock et al., 2011). Functional ability in performing activities of daily living was used as a primary outcome measure. Two trials were identified as meeting the inclusion criteria, which included a limited number of stroke patients (*n *=* *5) (Leigh, Burnstine, Ruff, & Kasmer, 1991; Strupp et al., 2003). In view of the limited number of trials identified and the limited number of stroke patients included, the authors recommended a wider review of interventions in acquired brain injury (ABI) populations. This Cochrane review is now on‐going (Rowe, Noonan, et al., 2014).

Further temporary intervention for ocular misalignment is botulinum toxin (BT) which has been reported widely in the literature for its use with strabismus (Rowe & Noonan, 2012). Its effects are reported to last for around three months. BT can also be helpful when planning a more permanent intervention such as ocular muscle surgery. Choudhuri, Sarvananthan, and Gottlob (2007) conducted a survey of neurologists and ophthalmologists across the UK regarding treatment preferences for nystagmus. The response rate could be viewed as low with 34% of neurologists and 37% of ophthalmologists returning the survey. Neurologists (60.8%) more commonly prescribed pharmaceutical agents as management options: Gabapentin and Baclofen were used most often.

#### 4.2.2 Substitutive treatment

Prisms are commonly used in clinical practice for the treatment and amelioration of the symptom of diplopia. Prisms may take the form of a temporary Fresnel prism or with a permanent prism ground into a spectacle lens. The theory of prisms is that the image of the object is shifted by a magnitude proportional to the strength of the prism, thus compensating for the eye misalignment (Firth & Whittle, 1994). The images are moved such that they overlap and allow the brain to fuse the images back to one image, in cases where the patient has potential for binocular single vision. Alternatively, the images are moved so they are separated to place the second image into a pre‐existing visual suppression area or, separated to an extent so that the second image can be ignored and/or is less troublesome for the patient.

In surveys of treatment provision for stroke survivors Pollock, Hazleton, & Brady (2011b) reported prisms to be the most common management provided (93%) followed by advice on head postures (64%) and convergence exercises (50%). Concurrently, Rowe, et al. (2013b) reported prisms and/or occlusion to be the most commonly prescribed intervention with the purpose to alleviate diplopia. A number of observational studies report the positive benefit of prisms and occlusion for relief of diplopia in stroke survivors (Rowe et al., 2011a). Furthermore, advice is frequently provided, primarily consisting of adaptive alternative head postures (AHPs) to avoid the direction of gaze associated with diplopia (Rowe et al., 2011a; Rowe et al., 2013b).

#### 4.2.3 Compensatory treatment

There are occasions when the use of prisms is not suitable, such as the deviation being too large and the presence of torsion or variable deviations (Firth & Whittle, 1994). In these circumstances occlusion can be used, which is frequently in the form of an opaque patch to eradicate the second image. Other options for occlusion include Bangerter foils or frosted tape which aim to blur the second image so it may be ignored (Hadid, Wride, Griffiths, Strong, & Clarke, 2008). It is also possible to provide partial sector occlusion for patients where diplopia is only bothersome in one direction of gaze (Routt, 2011). Furthermore, advice on compensatory strategies include adaptive head postures, reading options and the use of appropriate task lighting to optimize visual function (Rowe, et al., 2013b).

#### 4.2.4 Restitutive treatment

Conservative treatment options for specific ocular motility problems, such as convergence insufficiency, include vergence exercises (Rowe et al., 2011b). Improving ocular convergence with orthoptic vergence exercises can eliminate the symptom of diplopia and asthenopia in the near position (Adler, 2002). Rowe et al. (2009a) found reduced convergence of <10 cm was present in one third of stroke survivors which frequently contributed to reading difficulty.

Once recovery has ceased and if a deviation persists, a more permanent intervention may be considered, such as ocular muscle surgery. There are a variety of procedures for the many types of ocular motility conditions, which are detailed in the literature but are not specific to stroke populations. For example, one trial (Carruthers, Kennedy, & Bagaric, 1990) reported surgical success in 92.7% of adult participants receiving surgery for horizontal strabismus compared to 50.6% of those receiving BT after 6 months.

For cases of acquired nystagmus, relatively few ophthalmologists reported the use of surgical management (Choudhuri et al., 2007). For an overview of management options for nystagmus, including pharmacological, optical, surgical, and botulinum toxin, see Thurtell and Leigh (2010).

Although many of the treatment options for eye movement disorders have not been established within a stroke population specifically, the benefit would be much the same as with other cohorts. Furthermore, the lack of high quality clinical trials to determine the efficacy of treatments such as prisms and occlusion may not necessarily be required. It is well‐established that these treatments will alleviate the symptoms of diplopia without the need of clinical trials to prove so.

### 4.3 Central vision

Impaired central vision includes reduced visual acuity and contrast sensitivity. Pollock et al. (2012b) completed a Cochrane review investigating whether interventions used to treat other visual problems which are age related, also improved the functional outcome following stroke. In addition to stroke related visual problems, the authors also included patients with cataracts, glaucoma, age‐related macular degeneration, or diabetic retinopathy. They used functional ability as the primary outcome measure. Twenty four potential trials were found. However, it was not clear if these trials included stroke as a sub‐group. In view of this, the authors took the decision to exclude these trials as age‐related visual problems are already well‐covered by other Cochrane systematic reviews: age‐related macular degeneration (Casparis, Lindsley, Kuo, Sikder, & Bressler, 2012; Eandi, Giansanti, & Virgili, 2008; Evans, 2013; Evans & Lawrenson, 2012; Evans, Sivagnanaval, & Chong, 2010; Gehlbach, Li, & Hatel, 2012; Geltzer, Turalba, & Vedula, 2013; Giansanti, Eandi, & Virgili, 2009; Lawrenson & Evans, 2012; Parodi, Virgili, & Evans, 2009; Reddy & Krzystolik, 2006; Vedula & Krzystolik, 2008; Virgili & Bini, 2007; Williams, McKay, & Chakravarthy, 2014; Wormald, Evans, Smeeth, & Henshaw, 2007), cataracts (Alhassan, Kyari, & Ejere, 2008; Ang, Evans, & Metha, 2012; Calladine, Evans, Shah, & Leyland, 2012; Davison, Padroni, Bunce, & Rüschen, 2007; De Silva, Riaz, & Evans, 2014; Do, Gichuci, Vedula, & Hawkins, 2013; Fedorowicz, Lawrence, Gutierrez, & van Zuuren, 2011; Keay, Lindsley, Tielsch, Katz, & Schein, 2012; Mathew, Ervin, Tao, & Davis, 2012; Ong, Evans, & Allan, 2014; Riaz, de Silva, & Evans, 2013; Riaz, Mehta, Wormald, Evans, & Foster, 2006; Sivaprasad, Bunce, & Crosby‐Nwaobi, 2012), diabetic retinopathy (Grover, Li, & Chong, 2008; Lopes de Jesus, Atallah, Valente, & Moça Trevisani, 2008a; Lopes de Jesus, Atallah, Valente, & Moça Trevisani, 2008b; Smith & Steel, 2011; Virgili, Parravano, Menchini, & Brunetti, 2012), and glaucoma (Burr, Azuara‐Bianco, Avenell, & Tuulonen, 2012; Eldaly, Bunce, El Sheikha, & Wormald, 2014; Friedman & Vedula, 2006; Green, Wilkins, Bunce, & Wormald, 2014; Hatt, Wormald, & Burr, 2006; Kirwan, Rennie, & Evans, 2012; Law & Li, 2013; Minckler et al., 2006; Rolim de Moura, Paranhos, & Wormald, 2007; Sena & Lindsley, 2013; Simha, Braganza, Abraham, Samuel, & Lindsley, 2013; Vass et al., 2007; Waterman, Evans, Gray, Henson, & Harper, 2013; Wilkins, Indar, & Wormald, 2005). They recommended signposting readers to these Cochrane reviews covering different aspects of the specific conditions.

It is well‐recognized that many stroke survivors wore glasses prior to their stroke and it is important that they have access to their glasses, or receive a retest for glasses after their stroke (Lotery et al., 2000). For those patients who still have reduced central vision even with glasses correction, low visual aids (LVAs) such as magnifiers may be helpful (Rowe et al., 2011b). LVAs have been shown to be effective amongst patients suffering visual impairment for a variety of reasons, such as cataracts and macular degeneration. Information on reading aids such as electronic and non‐electronic optical aids, magnifiers and colored filters is available (Beasley & Davies, 2013; Virgili, Acosta, Grover, Bentley, & Giacomelli, 2013). A further systematic review addresses the use of low vision services, such as standard hospital‐based services, multidisciplinary services and services with an emphasis on the psychological needs of the patient (Binns et al., 2012). Further modifications to light and environment to aid visually impaired people at home include the use of color and contrast, avoiding clutter and using accessible appliances (Cooper, 2013; Joule, Levenson, & Brown, 2014). However, these have yet to be validated in the literature for their use in a stroke population.

Overall, advice and visual aids may be of benefit to stroke survivors with central visual impairment, however, these have not yet been evaluated within a specific stroke population. Further research is required to determine the benefit of these therapies following stroke.

### 4.4 Visual inattention / neglect

Unilateral visual inattention is the difficulty attending to one side of space (Bowen, Hazelton, Pollock, & Lincoln, 2013). A Cochrane review relating to spatial neglect following stroke focused on cognitive rehabilitation programs, encompassing a variety of bottom‐up and top‐down interventions (Bowen et al., 2013). Measures of functional ability / disability as a primary outcome measure were used. Twenty‐three trials were identified as meeting the inclusion criteria, eleven of which were new to this update (Cherney, Halper, & Papachronis, 2002; Cottam, 1987; Edmans, Webster, & Lincoln, 2000; Fanthome, Lincoln, Drummond, & Walker, 1995; Ferreira, Leite Lopes, Luiz, Cardoso, & André, 2011; Fong et al., 2007; Kalra, Perez, Gupta, & Wittink, 1997; Kerkhoff et al., 2012; Luukkainen‐Markkula, Tarkka, Pitkanen, Sivenius, & Hamalainen, 2009; Mizuno et al., 2011; Nys, Seurinck, & Dijkerman, 2008; Polanowska, Seniow, Paprot, Leniak, & Czonkowska, 2009; Robertson, 1990; Robertson, McMillan, MacLeod, Edgeworth, & Brock, 2002; Rossi et al., 1990; Rusconi, Meinecke, Sbrissa, & Bernardini, 2002; Schroder, Wist, & Homberg, 2008; Tsang, Sze, & Fong, 2009; Turton, O'Leary, Gabb, Woodward, & Gilchrist, 2010; Weinberg et al., 1977; Welfringer, Leifert‐Fiebach, Babinsky, & Brandt, 2011; Wiart et al., 1997; Zeloni, Farne, & Baccini, 2002). Meta‐analyses showed no significant persistent effect either on standardized assessments or for functional ability.

#### 4.4.1 Substitutive treatment

Menon‐Nair, Korner‐Bitensky, & Ogourtsova (2007) conducted a survey of Occupational Therapists in Canada asking what rehabilitation they perform for unilateral spatial neglect. The most commonly used interventions were perceptual retraining (33.2%) and visual scanning training (16.2%). No details were collected on how these interventions were performed. A subsequent survey engaged Orthoptists working in stroke care in Scotland and reported a high proportion would provide advice or explanation of neglect (72%). Other methods included typoscopes, reading aids, non‐computerized scanning therapy and onward referral to other professionals, although these methods were issued less frequently (21%) (Pollock, Hazleton, & Brady (2011b).

A subsequent trial (Machner et al., 2012) examined the effect of hemifield eye patching and optokinetic stimulation (OKS). This treatment was described as a “forced‐use” therapy comprising of sector occlusion over the non‐neglecting side of plano lenses and removed when completing the OKS. The results showed that both the control group and those receiving treatment had an equal improvement in neglect‐related functional disability over time.

#### 4.4.2 Compensatory treatment

A survey of Occupational Therapists (Pollock, Hazelton, & Brady, 2011a) reported a high proportion delivered treatment for visual neglect (89%) and visual field defects (69%), most commonly non‐computerized scanning training, activities of daily living training and provision of aids and modifications. Other compensatory methods of rehabilitation of visual neglect / inattention include occlusion and prism adaptation (Beis, André, Baumgarten, & Challier, 1999; Datié et al., 2006).

A Cochrane review meta‐analysis initially showed cognitive rehabilitation to have a significant immediate effect on standardized assessments (Bowen et al., 2013). The analysis was repeated with only high quality trials included. This significant effect was not maintained. In addition, trials which compared cognitive rehabilitation with visual scanning therapies were too heterogeneous to enable the authors to draw conclusions. In view of these findings the authors could not support or refute the interventions covered by the review. The recommendations were that clinicians should continue to follow national guidelines until further high quality evidence is available.

A further trial aimed to investigate whether or not smooth pursuit therapy is superior to standard scanning therapy (Kerkhoff et al., 2013). The authors reported more improvement following smooth pursuit training in both auditory and visual outcomes. These improvements were also seen for both mild and severe degrees of neglect with stability of improvement up to two weeks following training.

A variety of treatments have been described for visual neglect/ inattention after stroke, with compensatory scanning therapies appearing most favorable. However, due to lack of high quality evidence, these treatments cannot be recommended in clinical guidelines at present.

### 4.5 Visual perceptual deficits

Visual neglect/inattention is the most frequently occurring visual perceptual disorder following stroke (Hepworth et al., 2016). Additional deficits include visual hallucinations, object agnosia, color detection problems, and difficulty judging depth (Rowe et al., 2009b). Spontaneous recovery may occur for perceptual deficits. However, patients reported a benefit from verbal advice and coping strategies, as well as the relief associated with diagnosis and recognition of the impairment which can cause significant distress to the patient. A Cochrane review reported on the interventions for perceptual disorders following stroke (Bowen, Knapp, Gillespie, & Nicolson, 2011), however, the relevant papers for this review have been extracted and discussed elsewhere (Edmans et al., 2000; Mazer et al., 2003).

Interventions for perceptual deficits are often reported as case studies or small retrospective cohorts. One prospective observational study used cross‐modal word recognition training with a group of patients with pure alexia, which involved single words presented visually and via audio simultaneously. The group of patients were reported to read words from the training program quicker than untrained words, especially for the longer words. There was no transfer following training to letter or sentence reading. The improvement seen with words in the training program was not maintained at the follow‐up visit at two to four weeks after training had finished (Woodhead et al., 2013).

A range of visual perceptual disorders can occur following stroke however, very few treatments for these have been discussed in the current literature. It is possible that a number of treatments including advice are being used in practice with no clear evidence base and as such, further research is required to establish these treatments.

## 5 Conclusion

Overall, the findings from this review highlight implications for further research. There is a strong requirement for further high quality randomized controlled trials to determine the effectiveness of interventions when treating post‐stroke visual impairments. Furthermore, the majority of studies included in this review used a small number of patients in their study populations. Future research must address these issues and should consider the impact of interventions.

It is important to note that some interventions have been tested on broader populations and not an isolated stroke survivor population. However, in many visual conditions, the evidence can be applied to stroke survivors; for example, prisms have been shown to be effective in a general diplopia population and are an accepted and effective treatment.

The focus of future research should be relevant to activities of daily living, visual function, and vision‐related quality of life. Studies should aim to include long‐term follow‐up of the stroke survivors being offered visual treatment in order to accurately capture the effectiveness of these interventions and the transferability of these skills to activities of daily living. The current reported research has touched on recently developed, free web‐based treatments for visual search training and improving reading speeds. However, there is limited literature on these treatments and more research, preferably with control groups and larger population sizes, are required to investigate the effectiveness of these treatments further. Moreover, there are various treatment options currently used in clinical and social care to aid post‐stroke visual impairments such as environmental and lighting modifications, vertical reading, line guides, and typoscopes. These have yet to be thoroughly investigated. It is imperative that these treatments be addressed and evaluated in future studies to establish their effectiveness and provide an evidence‐base to inform clinicians and health professionals of all treatment options available.

## 6 Conflicts of Interest

The authors have no conflicts of interest to declare.

## 8 Disclaimer

The views expressed are those of the authors and not necessarily those of the NHS, the NIHR, or the department of health.
