# Supplementary material for: The treatment methods for post‐stroke visual impairment: A systematic review
Source: Brain Behav. 2017 Apr 6;7(5):e00682. doi: 10.1002/brb3.682 (PMC5434187; doi:10.1002/brb3.682)
Supplement: Supplementary file 1 [file BRB3-7-e00682-s001.docx]

| ***Supplemental table S1: Quality appraisal for papers using the CONSORT checklist*** | | | | | | | | | | | | | | | | | | | | | | | | | | | | | | | |
| --- | --- | --- | --- | --- | --- | --- | --- | --- | --- | --- | --- | --- | --- | --- | --- | --- | --- | --- | --- | --- | --- | --- | --- | --- | --- | --- | --- | --- | --- | --- | --- |
|  | Introduction | Methods | | | | | | | | | | | | | | | | Results | | | | | | | | | | Discussion | | | Other Info |
|  | Objectives | Trial design | Changes to methods | Eligibility | Interventions for each group | Outcome measures | changes to trail outcomes | Sample size | Interim analysis | Method of random allocation sequence | Randomisation | Implementation of random allocation | Generation of random allocation | Blinding | Similarity of interventions | Statistical methods | Additional analyses | No. of participants | Losses and exclusions | Dates of recruitment - follow up | Reason trial ended | baseline demographic | Analysis of original assigned groups | Results with precision | Binary outcomes | Additional analysis | Harms | Limitations | Generalisability | Consistent interpretation | Access to full protocol |
|  | 2b | 3a | 3b | 4a | 5 | 6a | 6b | 7a | 7b | 8a | 8b | 9 | 10 | 11a | 11b | 12a | 12b | 13a | 13b | 14a | 14b | 15 | 16 | 17a | 17b | 18 | 19 | 20 | 21 | 22 | 24 |
| Aimola et al. 2014 (43) | + | + | - | + | + | + | + | + | n/a | + | + | + | - | + | n/a | + | + | + | + | + | - | + | + | + | n/a | + | - | + | + | + | + |
|  | 2b | 3a | 3b | 4a | 5 | 6a | 6b | 7a | 7b | 8a | 8b | 9 | 10 | 11a | 11b | 12a | 12b | 13a | 13b | 14a | 14b | 15 | 16 | 17a | 17b | 18 | 19 | 20 | 21 | 22 | 24 |
| Beasley et al. 2013 (135) | + | + | - | + | + | + | + | + | n/a | + | + | - | - | + | n/a | + | + | + | ? | + | - | ? | + | + | + | + | - | + | + | + | - |
| Beis et al. 1999 (21) | + | + | - | + | + | + | - | - | n/a | - | + | - | - | - | n/a | + | + | + | ? | + | - | - | - | - | - | + | - | + | + | + | - |
| Bowers et al. 2014 (57) | + | + | - | + | + | + | - | + | n/a | + | + | + | + | + | + | + | + | + | + | + | - | - | + | + | + | + | - | + | + | + | + |
|  | 2b | 3a | 3b | 4a | 5 | 6a | 6b | 7a | 7b | 8a | 8b | 9 | 10 | 11a | 11b | 12a | 12b | 13a | 13b | 14a | 14b | 15 | 16 | 17a | 17b | 18 | 19 | 20 | 21 | 22 | 24 |
| Kerkhoff et al. 2013 (165) | + | + | - | + | + | + | - | + | n/a | + | + | + | + | - | - | + | + | + | + | + | - | + | + | + | + | + | - | + | + | + | - |
| Machner et al. 2014 (162) | + | + | n/a | + | + | + | - | + | n/a | + | + | + | + | n/a | + | + | + | + | + | + | - | + | + | + | + | + | - | + | + | + | - |
| Mazer et al. 2003 (38) | + | + | + | + | + | + | n/a | + | + | + | + | - | ? | + | + | + | + | + | + | + | - | - | + | + | + | + | + | + | + | + | + |
| Plow et al. 2012 (34) | + | + | - | + | + | + | - | + | n/a | - | - | - | - | + | + | + | + | + | + | ? | - | + | + | + | + | + | + | + | - | + | - |

= Not reported = Unclear = Reported

+

?

-
