# Supplementary material for: The treatment methods for post‐stroke visual impairment: A systematic review
Source: Brain Behav. 2017 Apr 6;7(5):e00682. doi: 10.1002/brb3.682 (PMC5434187; doi:10.1002/brb3.682)
Supplement: Supplementary file 2 [file BRB3-7-e00682-s002.docx]

***Supplemental table S2: Quality appraisal of papers using the GRACE checklist***

|  |  |  | | |  | |  | |  | |  | |  | |  | |  |  |  |  |
| --- | --- | --- | --- | --- | --- | --- | --- | --- | --- | --- | --- | --- | --- | --- | --- | --- | --- | --- | --- | --- |
|  | | | Data | | | | | | | | | | | Methods | | | | | | |
|  |  |  | Treatment | Primary outcomes | | Primary clinical outcome | | Validation | | Outcome | | Both groups measured equally | | Population restriction | | Comparison groups | | Confounding variables | Immortal-time bias | Analyses |
|  | | | D1 | D2 | | D3 | | D4 | | D5 | | D6 | | M1 | | M2 | | M3 | M4 | M5 |
| Datie et al. 2006 (164) | | | + | + | | + | | + | | + | | + | | ? | | + | | + | - | + |
| Jacquin-Courtois et al. 2013 (40) | | | + | + | | + | | + | | + | | + | | ? | | + | | + | - | + |
| Nelles et al. 2001 (4) | | | + | + | | + | | + | | + | | + | | ? | | - | | + | - | + |

-

= Not reported = Unclear = Reported

+

?
