# Supplementary material for: The treatment methods for post‐stroke visual impairment: A systematic review
Source: Brain Behav. 2017 Apr 6;7(5):e00682. doi: 10.1002/brb3.682 (PMC5434187; doi:10.1002/brb3.682)
Supplement: Supplementary file 3 [file BRB3-7-e00682-s003.docx]

***Supplemental table S3: Quality appraisal of papers using the PRISMA checklist***

|  | Title | Abstract | Introduction | | Methods | | | | | | | | | | | | Results | | | | | | | | Discussion | | Funding |
| --- | --- | --- | --- | --- | --- | --- | --- | --- | --- | --- | --- | --- | --- | --- | --- | --- | --- | --- | --- | --- | --- | --- | --- | --- | --- | --- | --- |
|  |  |  | Rational | Questions | Existing review protocol | Study Characteristics | Sources | Search strategy | Study selection | Data extraction | Variables | Risk of Bias | Summary measures | Data handling | Risk of Bias (cumulative) | Additional analyses | No. of studies | Characteristics data extraction | Risk of Bias (individual) | Benefits or Harms | Meta-analyses | Risk of Bias (across studies) | Additional analyses | Summary | Limitations | Generalisability |  |
|  | 1 | 2 | 3 | 4 | 5 | 6 | 7 | 8 | 9 | 10 | 11 | 12 | 13 | 14 | 15 | 16 | 17 | 18 | 19 | 20 | 21 | 22 | 23 | 24 | 25 | 26 | 27 |
| Bowen et al. 2013 (139) | + | + | + | + | - | + | + | + | + | + | + | + | + | + | + | + | + | + | + | + | n/a | + | + | + | - | + | + |
| Pollock et al. 2011 (70) | + | + | + | + | - | + | + | + | + | + | + | + | + | + | + | + | + | + | + | + | + | + | + | + | + | + | - |
| Pollock et al. 2011 (22) | + | + | + | + | - | + | + | + | + | + | + | + | + | + | + | + | + | + | + | + | n/a | + | n/a | + | + | + | - |
| Pollock et al. 2012 (85) | + | + | + | + | - | + | + | + | + | + | + | + | + | + | + | + | + | n/a | n/a | n/a | n/a | n/a | n/a | + | + | + | - |

+

?

-

= Not reported = Unclear = Reported
