# Supplementary material for: The treatment methods for post‐stroke visual impairment: A systematic review
Source: Brain Behav. 2017 Apr 6;7(5):e00682. doi: 10.1002/brb3.682 (PMC5434187; doi:10.1002/brb3.682)
Supplement: Supplementary file 4 [file BRB3-7-e00682-s004.docx]

| ***Supplemental table S4: Quality appraisal of papers using the STROBE checklist*** | | | | | | | | | | | | | | | | | |
| --- | --- | --- | --- | --- | --- | --- | --- | --- | --- | --- | --- | --- | --- | --- | --- | --- | --- |
|  | Methods | | | | | | | | | Results | | | | | Discussion | | |
|  | Study design | Participants | Variables | Data source | Bias | Study size | Quantitative variables | Statistical methods | Participants | Descriptive data | Outcome data | Main results | Other analyses | Key results | Limitations | Interpretation | Generalisability |
|  | 4 | 6 | 7 | 8 | 9 | 10 | 11 | 12 | 13 | 14 | 15 | 16 | 17 | 18 | 19 | 20 | 21 |
| Bergsma et al. 2012 (67) | - | + | + | + | + | + | + | - | + | + | + | - | n/a | + | + | + | + |
| Choudhuri et al. 2007 (75) | + | + | + | + | - | + | - | - | + | + | + | + | + | + | - | + | n/a |
| Freeman & Rudge. 1987 (10) | + | + | + | + | - | + | - | - | + | + | + | - | n/a | + | - | + | - |
| Gall et al. 2012 (66) | + | + | + | + | + | + | + | + | + | + | + | + | n/a | + | + | + | + |
| Giorgi et al. 2009 (56) | + | + | + | + | + | + | + | + | + | + | + | + | - | + | - | - | - |
| Hayes et al. 2012 (47) | + | + | + | + | + | - | - | - | + | + | + | n/a | n/a | + | + | + | + |
| Lane et al. 2010 (41) | + | + | + | + | + | - | + | + | + | + | + | + | + | + | - | + | + |
| Lotery et al. 2000 (133) | + | + | ? | + | - | + | - | - | + | + | + | + | n/a | + | - | + | + |
| Mannan et al. 2010 (46) | + | + | + | + | + | - | + | + | + | + | + | + | n/a | + | - | + | + |
| Marshall et al. 2010 (68) | + | + | + | + | + | - | + | + | + | + | + | - | n/a | + | + | + | + |
| Menon-Nair et al. 2007 (161) | + | + | + | + | + | + | + | + | + | + | + | + | n/a | + | + | + | + |
| Mueller et al. 2007 (60) | + | + | + | + | + | + | + | + | + | + | + | + | + | + | + | - | - |
| Nelles et al. 2010 (48) | + | + | + | + | + | - | + | + | - | + | + | - | n/a | + | - | + | - |
| Ong et al. 2012 (45) | + | + | + | + | + | + | + | + | + | + | + | + | n/a | + | + | + | + |
| Ong et al. 2015 (44) | + | + | + | + | - | + | + | + | + | + | + | + | n/a | + | + | + | + |
| Pambakian et al. 2004 (42) | + | + | + | + | - | + | - | + | + | + | + | + | n/a | + | - | + | + |
| Poggel et al. 2007 (61) | + | + | + | + | + | + | + | + | + | + | + | + | n/a | + | + | + | + |
| Pollock et al. 2011 (163) | + | + | + | + | + | + | + | n/a | + | + | + | + | n/a | + | - | + | + |
| Pollock et al. 2011 (36) | + | + | + | + | + | + | + | n/a | + | + | + | + | n/a | + | + | + | + |
| Reinhard et al. 2005 (65) | + | + | + | + | + | - | + | + | + | + | + | + | n/a | + | + | + | + |
| Romano et al. 2008 (62) | + | + | + | + | + | + | + | + | + | + | + | + | n/a | + | + | + | + |
| Rowe & VIS 2009 (13) | + | + | + | + | + | + | + | + | + | + | + | + | n/a | + | - | + | + |
| Rowe &VIS 2011 (37) | + | + | + | + | + | + | + | + | + | + | + | + | n/a | + | + | + | + |
| Rowe &VIS 2011 (78) | + | + | + | + | + | + | + | + | + | + | + | + | n/a | + | + | + | + |
| Rowe &VIS 2011 (77) | + | + | + | + | + | + | + | + | + | + | + | + | n/a | + | + | + | + |
| Rowe &VIS 2011 (81) | + | + | + | + | + | + | + | + | + | + | + | + | n/a | + | + | + | + |
| Sabel et al. 2004 (63) | + | + | + | + | + | + | + | + | + | + | + | + | + | + | - | + | + |
| Sabel et al. 2013 (64) | + | + | + | + | + | + | - | + | + | + | + | + | n/a | + | - | + | + |
| Schmielau & Wong 2007 (59) | + | + | + | + | + | + | + | + | + | + | + | + | + | + | ? | + | + |
| Woodhead et al. 2013 (19) | + | + | + | + | + | - | + | + | ? | + | + | + | n/a | + | - | + | + |
| Zihl 1995 (49) | + | + | + | + | + | - | + | + | ? | + | + | + | n/a | + | - | + | - |
| Zihl & von Cramon 1979 (20) | - | + | + | + | - | - | - | - | - | + | + | + | n/a | + | - | - | - |
| Zihl & von Cramon 1982 (58) | - | ? | + | + | + | - | - | - | ? | + | + | + | n/a | ? | - | + | + |
| Zihl & von Cramon 1985 (39) | + | + | + | + | + | + | - | - | ? | + | + | + | n/a | + | + | + | + |

= Not reported = Unclear = Reported

+

?

-
